# Multi-Party Cryptographic Key Distribution Protocol over a Public Network Based on a Quick-Response Code

**DOI:** 10.3390/s22113994

**Published:** 2022-05-25

**Authors:** Wen-Kai Yu, Ying Yang, Ya-Xin Li, Ning Wei, Shuo-Fei Wang

**Affiliations:** 1 Center for Quantum Technology Research, School of Physics, Beijing Institute of Technology, Beijing 100081, China; 3120201526@bit.edu.cn (Y.Y.); 3120181429@bit.edu.cn (Y.-X.L.); 3120201518@bit.edu.cn (N.W.); 3120195769@bit.edu.cn (S.-F.W.); 2 Key Laboratory of Advanced Optoelectronic Quantum Architecture and Measurement of Ministry of Education, School of Physics, Beijing Institute of Technology, Beijing 100081, China

**Keywords:** cryptographic key distribution, multi-party communication, computational ghost imaging, quick-response code, watermark embedding and extraction, identity authentication

## Abstract

In existing cryptographic key distribution (CKD) protocols based on computational ghost imaging (CGI), the interaction among multiple legitimate users is generally neglected, and the channel noise has a serious impact on the performance. To overcome these shortcomings, we propose a multi-party interactive CKD protocol over a public network, which takes advantage of the cascade ablation of fragment patterns (FPs). The server splits a quick-response (QR) code image into multiple FPs and embeds different “watermark” labels into these FPs. By using a CGI setup, the server will acquire a series of bucket value sequences with respect to different FPs and send them to multiple legitimate users through a public network. The users reconstruct the FPs and determine whether there is an attack in the public channel according to the content of the recovered “watermark” labels, so as to complete the self-authentication. Finally, these users can extract their cryptographic keys by scanning the QR code (the cascade ablation result of FPs) returned by an intermediary. Both simulation and experimental results have verified the feasibility of this protocol. The impacts of different attacks and the noise robustness have also been investigated.

## 1. Introduction

In the information age, people’s lives are inseparable from the Internet, and information security has become one of the most critical issues. Especially after the outbreak of COVID-19, online work, meetings and payment have become frequent. The public network brings convenience to people but also has various security risks. As we know, the guarantee of information security relies on the reliable cryptosystems. With the rapid development of optical information technology, many optical encryption schemes have been proposed. For example, Refregier et al. [1] proposed a double random phase encoding (DRPE) scheme in 1995 to encode information by using the phase characteristic of light, and this scheme has evolved into many variants [2,3,4,5]. In the same year, the idea of ghost imaging (GI) was proposed by Pittman et al. [6]. It was first experimentally demonstrated with quantum entangled photon pairs [6] and later extended to true thermal light [7], pseudothermal light [8], X-ray [9] and particles [10,11,12]. Later research found that it can also be simplified from double-arm to single-arm by using a spatial light modulator (SLM) to perform optical encoding, which is called computational ghost imaging (CGI) [13]. Combined with compressed sensing (CS), the qualities of ghost images can be greatly improved [14]. Since GI generally uses completely random patterns to encode the object image, it has been successfully used for optical encryption [15,16,17].

It is not enough to just encrypt information, as encryption allows a certain error rate, since even if some part of the encrypted information is missing in the transmission process, it will not affect the overall content too much. If the cryptographic keys (CKs) themselves are distributed directly, any error will have a huge impact on the information to be encrypted, which requires higher security of its distribution. Thus, the cryptographic key distribution (CKD) is a hard nut to crack. In 1984, the famous BB84 protocol [18] was proposed to realize quantum key distribution (QKD). The QKD shows perfect security. Any eavesdropping can be detected because it has quantum mechanics as its theoretical support [19]. Presently though, quantum channels are still too expensive to be used in practice and are difficult to make compatible with traditional optical fiber networks. Furthermore, it is also hard for QKD protocols [18,19,20] to realize multi-party CKD. The generation of entangled light and single-photon measurements undoubtedly increase the complexity of the protocols, and impose high requirements on hardware devices, resulting in low key generation rates, high bit error rates, poor stability and low reliability for distributed CKs. Additionally, for long-distance transmission, expensive trusted quantum repeaters are required. Therefore, it is urgent to study a CKD protocol that can work with regular public channels and has the features of low cost, high efficiency and high security comparable with QKD.

In our previous work, we designed CGI-based multi-party CKD protocols [21,22] over a public network, where the modulated patterns are treated as pre-shared initial secret keys for later privacy amplification, the encrypted bucket values sampled by a CGI setup are sent to legitimate users through public channels. Each user can complete independent identity authentication via CS [21] and extract some digits after the decimal points of gray values in the images reconstructed by GI to form a random bit sequence (i.e., the distributed CK) [21,22]. Since the public network is used, remote transmission is not difficult. Beyond that, the CKD protocol can also be improved in many other ways. For instance, Yi et al. [23] proposed a camouflaged encryption method based on compressive GI, where the secret image is hidden in the camouflaged image to further improve the concealment of information. Later, they also proposed another hybrid encryption scheme based on temporal ghost imaging [24], which uses asymmetric public key cryptography to enhance security. Ye et al. [25] designed two novel generation schemes of pseudo-random patterns in the space-time dimension to increase the capacity of information embedding. In these studies, the interaction between multiple users is often ignored, which is worth researching and exploring.

In the above schemes, the information carrier is either the object image or bucket values; their information capacity is limited. To increase the information capacity, one needs to either increase the pixel-size of the image or the number of the modulated patterns. Sui et al. [26] proposed to an encryption scheme based on a customized data container, which is used as the encrypted image to enhance the ability to encrypt more information. Later, we proposed to build the mapping relationship of additive stitching images to be distributed and private key libraries to further increase the information capacity of the CKD protocol [27], but its susceptibility to attacks still needs to be improved. As we know, the quick response (QR) code has become a necessity in our daily lives, for it can be quickly identified by our smartphones and can store a great deal of information in the form of two-dimensional (2D) image encoding [28,29,30]. Besides, it also has high error tolerance capability. Barrera et al. [31,32] directly used the QR code as a container of the secret information for optical image encryption. By utilizing its high-contrast binary image property, one can acquire higher robustness performance against noise in encryption. After that, Zhao et al. [33] introduced the QR code into the CGI to enlarge the information capacity of optical encrypted signal. On the other hand, the watermarking can be used as a privacy protection technology that embeds labels in visible images and texts [34], or a data hiding technology that hides the useful information in imperceptible signals to increase robustness to attacks or undetectability. Due to its security characteristics, the watermarking has been widely used in cryptosystems. As in the public network CKD protocol, the bucket values transmitted through public channels can be further embedded with watermarks to further increase the security [35]. If we could absorb both the excellent information capacity of the QR code and security characteristics of the watermarking technology, we could definitely build a CKD protocol with superior performance.

In this work, we propose a multi-party interactive CKD protocol over a public network, which uses a QR code as the container of CKs and embeds “watermark” labels in the idle functional region of QR code image. In this protocol, the server splits the QR code image into multiple different fragment patterns (FPs), in each of which an independent image label that corresponds to each user is embedded in its unused functional region. By using a CGI setup, these FPs are separately encrypted into random bucket value sequences, which will be then sent to multiple legitimate users through public channels. This data hiding strategy also makes the watermark labels undetectable, confuses the audiovisual signal, increases the confidentiality and imperceptibility of the CKs and avoids content leakage. After receiving bucket values and performing image reconstruction, the receivers can conduct identity authentication and detect potential attacks, according to the content of their recovered watermark labels, and then send their results to a reliable intermediary for joint authentication. According to the cascade ablation result of FPs (complete QR code image) returned back from the intermediary, legitimate users can quickly extract their CKs by scanning the QR code. The cascade-ablation-based multi-party interactive identity authentication will improve the security of the protocol, the use of the QR code image increases the capacity of information and the watermarking technology is used for identity self-authentication and attack detection.

## 2. Protocol

**Figure 1:**
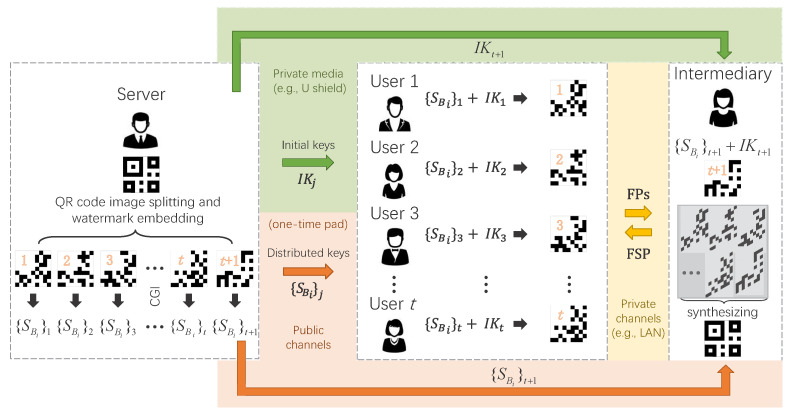
Schematic of a multi-party cryptographic key distribution (CKD) protocol over a public network based on a quick-response (QR) code. FPs: fragment patterns; FSP: fragment synthesis pattern.

As shown in Figure 1, this protocol can be divided into two parts: cryptographic key preparation and encrypted signal transmission over a public network; watermark-based identity authentication and cascade-ablation-based cryptographic key extraction.

**Part I**: Cryptographic key preparation and encrypted signal transmission over a public network.

**1. Sharing of initial keys.** The server generates *N* random binary patterns $IK_{j}$ (j=1,2,3,⋯,t+1) of the same pixel-size as the QR code image, and shares them to each legitimate user through an absolutely secure private medium (such as a non-reproducible flash disk or a U shield) in advance. Secure media such as USB flash drives or USB shields are easy to carry and suitable for storing large-scale initial keys. In view of this, the server can distribute such a non-reproducible medium to every legitimate user in advance to ensure absolute security.

**2. Preparation of FPs.** The QR code image encoded with secret information is regarded as the original image to be encrypted. As shown in Figure 1, the server first divides it into t+1 fragment patterns (FPs) following a cascade ablation principle. One FP is for the intermediary and *t* FPs are for *t* legitimate users.

**3. Watermark embedding.** The server embeds corresponding “watermark” labels in the fixed pixel regions of FPs (here the upper left corner of the FP is selected as the watermark-embedding position). The watermarking technology used here can be treated as a kind of privacy protection.

**4. Encrypted signal transmission over a public network.** The above t+1 2D FPs will be encrypted into t+1 one-dimensional (1D) measured bucket value vectors ${\left\{S_{B_{i}}\right\}}_{j}$ (j=1,2,3,⋯,t+1) via a CGI optical setup, and then be sent to legitimate users over a public network. The 2D FPs used here are all binary and can be generated numerically. An intuitive approach is to use the numerical model to calculate and generate the corresponding bucket values on a computer. However, a computer’s numerical simulation cannot generate true random numbers, which cannot meet the requirement of the CGI-based CKD protocol for true randomness. Thereby, it is necessary to introduce true random variables, and the optical setup is the best choice. As we know, the optical setup involves the true random fluctuations of the light source, the true random stray light, the true random variation of the illumination, the true random electrical shot noise of the detector, etc. These are all very good true random physical sources, which can provide the CKD protocol excellent security guarantees. Hence, the physical setup is very essential for our CKD protocol.

**Part II**: Watermark-based identity authentication and cascade-ablation-based cryptographic key extraction.

**5. GI reconstructions and attack self-checking.** By using intensity correlation functions [8], the legitimate users can quickly reconstruct ghost images of FPs from their received bucket values and pre-shared initial keys $IK_{j}$. After image binarization (by using smoothing-based or sorting-based strategy), the legitimate users can extract their “watermarks” for identity authentication. If the content in the recovered “watermark” is clear and regular, then the legitimate user can determine that the received data are safe.

**6. Watermark removal.** After each user finishes authentication, he/she will remove the “watermark” in the aforesaid fixed pixel region to get the binary result of FP.

**7. Cascade-ablation-based CK extraction.** All users send their FPs to a trusted intermediary through private channels (e.g., local area network (LAN)). Generally, the transmission over private channels is less vulnerable to attacks. As we know, the LAN is a closed network with small coverage and is isolated from the external network. It has extremely high security and is very suitable for small-scale short-term secure communication between the intermediary and users. Here, the intermediary synthesizes the received FPs and its own FP together by cascade ablation, i.e., performing cascade exclusive-or (XOR) operations on the values that fall into the same pixel positions as these FPs—$XOR(\cdots XOR\left(XOR\left(FP_{1},FP_{2}\right),FP_{3}\right),\cdots ,FP_{t+1})$—to acquire the final fragment synthesis pattern (FSP). This process plays an important role in affirming the legitimacy of all users. If this FSP turns out to be a readable QR code image, the joint authentication succeeds, and then this FSP (i.e., the recovered QR code) will be returned back to the legitimate users also through aforementioned private channels. Finally, the legitimate users can scan this QR code to obtain the CKs to be distributed.

Differently from traditional CGI-based CKD schemes, this protocol can detect attacks occurring in public channels in real time via the users’ watermark recognition. In addition, by using cascade ablation, it can also judge whether there is a fake among users who intends to interrupt the CKD process. This double insurance mechanism significantly enhances the security of the CGI-based CKD protocol.

Since the modulated patterns (i.e., the initial key) $IK_{j}$ are of large-scale and the whole protocol actually uses the mechanism of privacy amplification, each user’s patterns can be reused while the CKs acquired during one round of the CKD process can only be used once (following Vernam’s one-time pad idea). This is because the initial key distributed in advance for each user and intermediary contains *N* random binary patterns $IK_{j}$, which are fixed and can be reused for multiple rounds of CKDs and communications, and the number of rounds *M* can be much greater than *N*. Although the pixel-unit size of the QR code synthesized in each round is limited (of the same size as one random binary modulated pattern), it actually contains more information than its size, which essentially increases the capacity of the information. Additionally, M≫N rounds of CKDs will generate M≫N such QR codes, thereby finally realizing the effect of privacy amplification (or key growth).

## 3. Simulation and Experimental Results

**Figure 2:**
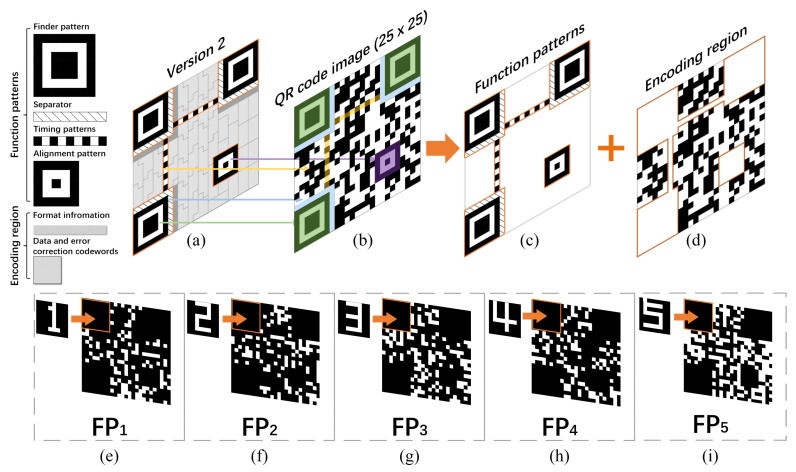
Schematic of QR code image segmentation and watermark embedding for the t=4 case. (**a**) QR Code (Version 2) barcode symbology specification; (**b**) QR code image of 25×25 pixel-units; (**c**) function patterns; (**d**) encoding region; (**e**–**i**) five fragment patterns (FPs).

**Figure 3:**
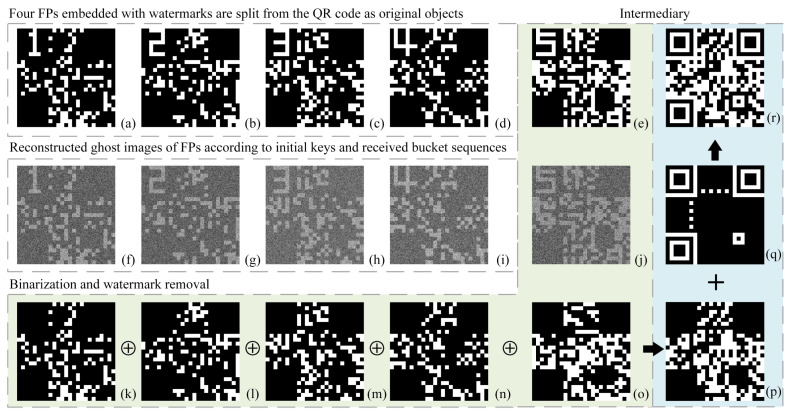
Simulation results for t=4 case. (**a**–**e**) Five watermarked FPs; (**f–j**) are the recovered ghost images; (**k**–**o**) five binary FPs with the watermarks being removed from (**f**–**j**); (**p**) the composite image synthesized from (**k**–**o**) by using the cascade ablation strategy; (**q**) the function patterns of a QR code of Version 2; (**r**) the sum of (**p**,**q**), i.e., the recovered QR code image.

Some numerical simulations were conducted to validate the feasibility of this protocol. As we all know, the QR code can encode string information into a 2D binary image by some rules, and its pixel-size increases with the string length. The string information to be encoded can be a link address (which can directly jump to a hyperlink after scanning the QR code and then present the images, videos, texts, web pages, etc.) or a sequence of pure characters. Here, for simplicity and without loss of generality, we directly set the encoding information of the QR code to an 18-byte disk address—“G:\01\001\0001\012”—which can be regarded as the actual CK to be distributed or the retrieval code of the CK. If it is a retrieval code, the legitimate user can go to the actual disk address on his/her flash disk (or U shield) where his/her CK library is stored in advance to extract the corresponding CK. By this means, it actually realizes the expansion of CKs. After encoding, we obtained a QR code of 25×25 pixel-units. To our knowledge, a QR code image of Version 2 consists of function patterns and encoding regions, as shown in Figure 2. We segmented the encoding region following the principle of cascade ablation. Taking t=4 as an example, five FPs of the same 25×25 pixel-units would be generated. In the upper left corner of each FP, we embedded an unique watermark for each legitimate user (for simplicity and without loss of generality, here we set the watermarks to be the users’ numbers), as shown in Figure 2e–i and Figure 3a–e. To reduce the influence of inevitable noise fluctuations, here we took an upsampling strategy (i.e., upsampling a low-resolution image to a higher resolution): we assumed that each pixel-unit of the FP was sampled by a 0–1 random matrix of ν×ν pixels in each modulation. Here, ν was set to 8; thus, the real sizes of both FPs and random binary modulated patterns $IK_{j}$ were 200×200 pixels. Then, the server would encrypt each FP (regarded as an original object image) into a bucket value sequence ${\left\{S_{B_{i}}\right\}}_{j}$ (j=1,2,3,4,5) via a CGI setup, which used $IK_{j}$ as random binary modulated patterns. The measured five sequences ${\left\{S_{B_{i}}\right\}}_{j}$ (j=1,2,3,4,5) were then sent to four legitimate users and an intermediary by public channels. After that, the legitimate users and the intermediary reconstructed their corresponding FPs according to the received bucket value sequences and pre-shared initial key $IK_{j}$. After performing binarization on these FPs, the legitimate users could extract their watermarks for the sake of identity authentication. If the self-authentication succeeded and the extracted watermark showed no abnormalities, then the user would remove the watermark content from the region of function patterns, as shown in Figure 3k–n, and sent the result to the intermediary through a private channel (e.g., a LAN). The intermediary then synthesized four received FPs with the watermarks being deleted and its own FP (see Figure 3o) to obtain a composite image by using the cascade ablation strategy. By adding the function patterns (see Figure 3q) to this composite image (Figure 3p), the final FSP (recovered QR code, as shown in Figure 3r) could be generated successfully. In the end, the intermediary sent this FSP back to each legitimate user also through the foregoing private channel (e.g., LAN).

**Figure 4:**
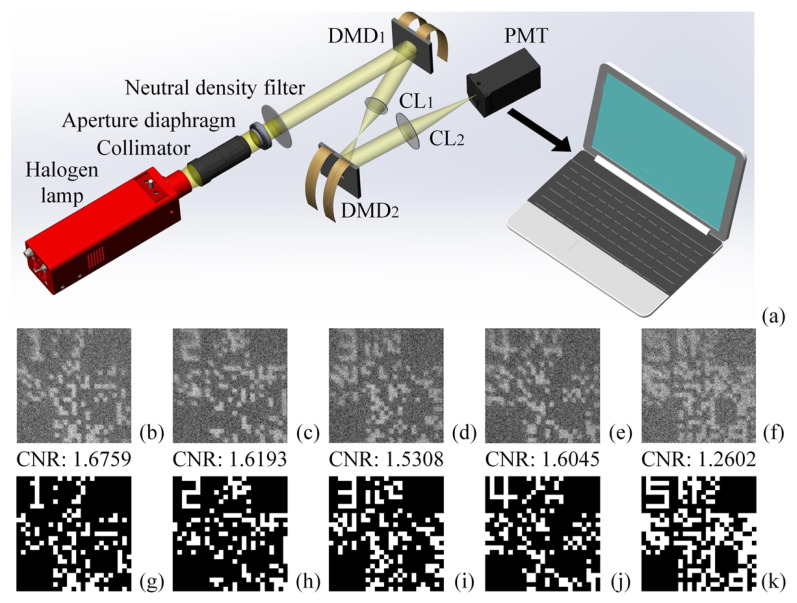
Experimental setup (**a**) and results (**b–k**). (**b**–**f**) Five recovered ghost images of 200×200 pixels (ν=8) with N= 40,000; (**g**–**k**) the binarized results of (**b**–**f**). DMD: digital micromirror device; CL: convergent lens; PMT: photomultiplier tube.

The experimental setup of CGI is given in Figure 4a. The thermal light emitted from a stabilized tungsten-halogen lamp was amplified, collimated and attenuated to form a parallel beam with a diameter close to the diagonal length of the first digital micromirror device’s (DMD) working plane. The light beam illuminated the first DMD, which was encoded with *N* random binary patterns of 200×200 pixels (also with ν=8). The reflected light from the first DMD passed through a convergent lens (CL) with a focal length of 50 mm and imaged onto the second DMD, which was loaded with FPs as original object images (a common practice in SPI [36,37,38]). Using another DMD rather than transparent films or etched plates to present FPs can facilitate the object switching without the need to change the light path and save costs. The reflected light of the second DMD was then focused onto a photomultiplier tube (PMT) (served as a bucket detector to record the total light intensities) through a CL also of 50 mm focal length. For a 4-user CKD case, the reconstructed ghost images of 5 FPs and their binarized results were presented in Figure 4b–f and 4g–k, respectively.

Two standards were applied to evaluate the quality of reconstructed images. One was the contrast-to-noise ratio (CNR): $\mathrm{CNR}\left(G\right)=\frac{\langle G(x_{in})\rangle -\langle G(x_{out})\rangle }{\sqrt{\frac{1}{2}\left[\Delta^{2}G\left(x_{in}\right)+\Delta^{2}G\left(x_{out}\right)\right]}}$, which was used as an assessment for the reconstructed grayscale images before binarization, where $\Delta^{2}G\left(x\right)=\langle G{\left(x\right)}^{2}\rangle -{\langle G(x)\rangle }^{2}$ denotes the variance; $\langle \cdot \rangle$ represents the ensemble average operator; $x_{in}$ and $x_{out}$ stand for the pixels inside and outside the transmitted object regions [39], respectively. The larger the CNR value, the better the quality of reconstructed image. Another criterion we used was the number of wrong points (i.e., bright pixel-units were misjudged as dark pixel-units or dark pixel-units were misjudged as bright pixel-units) existing in one binarized FP result compared with the original FP, which can intuitively indicate the correctness of this binarized FP.

**Figure 5:**
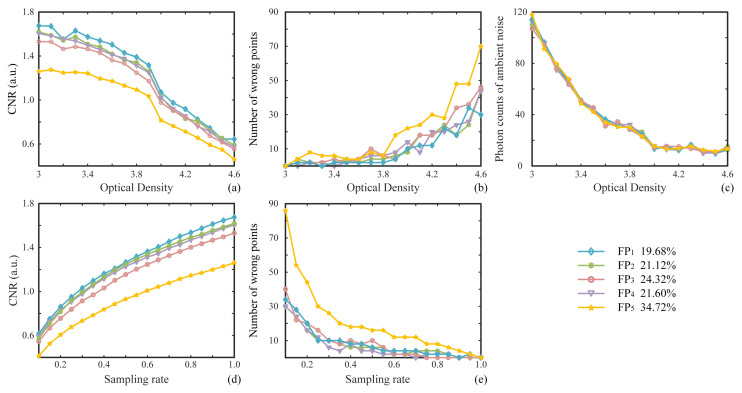
Correctness analysis of the binarized FPs. (**a**,**b**) The variation trends of contrast-to-noise ratios (CNRs) and the number of wrong points with the increase in the attenuation coefficient optical density (OD), corresponding to the five recovered results, respectively. (**c**) The photon counts of background noise as a function of the OD value. (**d**,**e**) The trends of CNRs and the number of wrong points with the increase in the sampling rate. The corresponding ratios of the numbers of ones in five original FPs to the total 25×25 pixel-units are listed in the legend.

Figure 5 shows the performance analysis of the protocol with the changes in the optical density (OD) of the used neutral density filter (NDF) and sampling rate. The OD can be treated as an attenuation coefficient which is defined as $\mathrm{OD}=\log_{10}\left(\frac{1}{T}\right)$, where *T* denotes the transmittance. From Figure 5a we can see that the CNRs of the restored FPs have a continuous downward trend with the increase in the OD. Additionally, Figure 5b shows that when the OD is less than 3.5, the number of wrong points can be kept at a relatively low level. When the OD is greater than 3.5, the number of wrong points increases with the OD value. As far as we know, the value of OD determines the level of the total light intensity that enters into the PMT. Generally, the larger the OD value is, the more severely the photon counts of both signal and ambient noise will be attenuated. However, we can see from Figure 5c that, as the OD value increases, the attenuation of photon counts of ambient noise (with double DMDs being encoded with all-zero matrices) will tend toward saturation. The photon counts of the signal are usually larger than those of the ambient noise, and the signal attenuation saturation occurs latter than the noise attenuation saturation; thus, the signal attenuation amplitude is larger than the noise amplitude in high ODs, which finally leads to a decrease in the measured signal-to-noise ratio and an increase in the number of wrong points in the case of high OD values. Figure 5d,e shows the trends of CNRs and the number of wrong points with the increase in the sampling rate: the CNR increases and the number of wrong points becomes smaller as the sampling rate increases. In Figure 5e we can see that full sampling is the minimum sampling rate for acquiring perfect FPs and final QR results without wrong points by using a second-order intensity correlation. In the legend of Figure 5, we give the sparsity ratios of the number of the pixel-units with their values being ones to the total 25×25 pixel-units in each FP. In Figure 5a,b and 5d,e, we can see that the highest sparsity ratio ($FP_{5}$ as represented by the yellow curves) always generates the poorest results (the lowest CNRs and the largest number of wrong points), and the quality of restored result is inversely proportional to the sparsity ratio in all cases. Thus, to ensure each user can reconstruct the FP with absolute accuracy, the sparsity ratio in each fragment needs to be set within a reasonable range.

## 4. Attack Detection and Security Analysis

**Figure 6:**
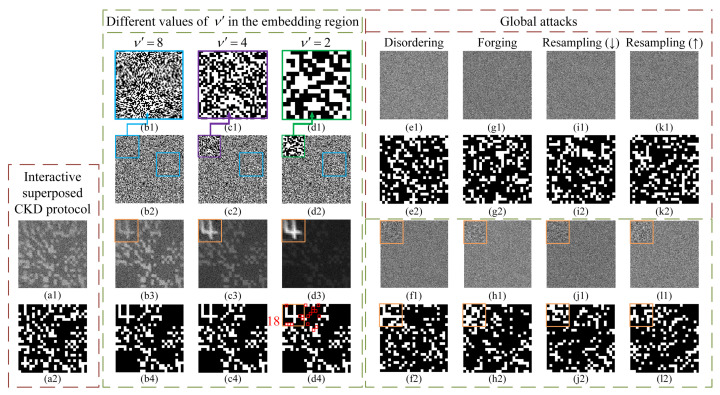
Results of two CKD protocols based on computational ghost imaging (CGI) under different ν’ values and different global attacks. (**a1**,**a2**) The recovered ghost image and binarized result of $FP_{random}$ by using the interactive superposed CKD protocol. (**b1**–**b4**,**c1**–**c4**,**d1**–**d4**) are the enlarged watermark-embedding regions of the modulated patterns; the complete matrices of these patterns; and recovered ghost images of $FP_{4}$ and their binarized results, acquired using the proposed protocol, with different ν’ values in the watermark-embedding region—8, 4 and 2, respectively. (**e1**,**e2**,**g1**,**g2**,**i1**,**i2**,**k1**,**k2**) in the brown dotted box and (**f1**,**f2**,**h1**,**h2**,**j1**,**j2**,**l1**,**l2**) in the green dotted box are the restored ghost images and their binarized images corresponding to the interactive superposed CKD protocol and our protocol under different global attacks (disordering, forging, resampling (sub-resampling) and resampling (over-resampling)), respectively.

Next, we analyze the role of the watermark-embedding region of the FP on attack detection. For a fair comparison, we picked a CGI-based CKD protocol [27] that is the most similar to this proposed protocol in the recent literature and acquired its experimental results as a reference. This recently developed protocol also generates different FPs (mutually exclusive) for multiple legitimate users and determines whether there exists an attack by checking whether the superposed result of FPs (by simple addition) recovered by users is a regular pattern. However, it does not use the watermarking technology; it cannot tell which channel the attack occurred in. Let us call it the interactive superposed CKD protocol. It should be mentioned that its synthesized regular image does not have a functional region as in the QR code image, so the overall pixel resolution of modulated patterns used for it should be consistent and each FP should be purely random. We took $FP_{4}$ as an example and directly filled the functional region of $FP_{4}$ with 0–1 random speckles to form a certain FP of the interactive superposed CKD protocol, denoted as $FP_{random}$. Since the FP in our protocol has a watermark-embedding region and non-watermark-embedding region, we specially designed the following experiment by setting different spatial resolutions for these two regions. In this experiment, we set the image size of both $FP_{4}$ and $FP_{random}$ to 25×25 pixel-units. In the above, we assumed that each pixel-unit of the FP was sampled by a 0–1 random matrix of ν×ν pixels in each modulation. We set the values of ν in the entire modulated patterns with respect to $FP_{random}$ and those in the non-watermark-embedding region of the modulated patterns with respect to $FP_{4}$ to 8, and we set the values of ν’ in the watermark-embedding region of $FP_{4}$ to 8, 4 and 2. The reconstructed ghost image and corresponding binarized result (showing no wrong points) of the interactive superposed CKD protocol are given in Figure 6a1,a2. The enlarged watermark-embedding regions of the modulated patterns for $FP_{4}$ and their complete matrices are presented in Figure 6b1–d1 and 6b2–d2, respectively. In Figure 6b3–d3, we provide the corresponding recovered ghost images of $FP_{4}$ under full sampling (i.e., N= 40,000). In ν’=8 and ν’=4 cases, there were no wrong points in binarized results of $FP_{4}$ (see Figure 6b4,c4). In the binarized image shown in Figure 6d4, the number of wrong points increased dramatically, and there were seven wrong points in the watermark-embedding region of 8×8=64 pixel-units and 11 wrong points in the non-watermark-embedding region of 25×25−8×8=561 pixel-units. Thereby, it is easy to find that the percentage of wrong points in the watermark-embedding region (7/64 = 10.9375%) is much greater than that in the non-watermark-embedding region (11/561 = 1.9608%). Additionally, in non-watermark-embedding region, the wrong points always concentrated around the watermark-embedding region. This is because as the watermark in the embedding region becomes brighter, the contrast of the bright and dark pixels in the non-watermark-embedding region becomes worse (see Figure 6b3–d3), which will inevitably lead to some misjudgments in the binarization process, especially in and around the watermark-embedding region. It is worth mentioning that, according to practical needs, we can arbitrarily adjust the values of ν and ν’ in these two regions. Additionally, based on the above results, in the following tests we set ν’ in the watermark-embedding region and ν in non-watermark-embedding region to 4 and 8, respectively.

**Figure 7:**
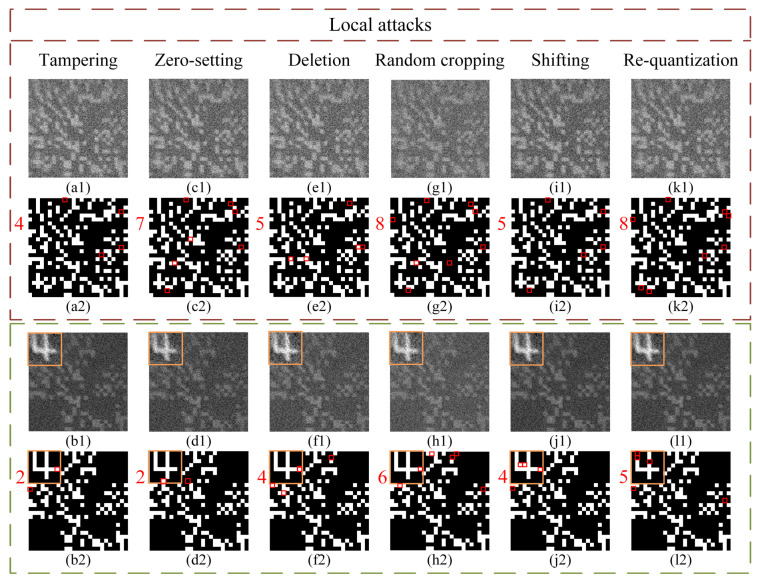
Results under six different kinds of local attacks. (**a1**,**a2**,**c1**,**c2**,**e1**,**e2**,**g1**,**g2**,**i1**,**i2**,**k1**,**k2**) in the brown dotted box and (**b1**,**b2**,**d1**,**d2**,**f1**,**f2**,**h1**,**h2**,**j1**,**j2**,**l1**,**l2**) in the green dotted box are the recovered ghost images and their binarized results obtained using the interactive superposed CKD protocol and the proposed protocol under six different types of local attacks: tampering, zero-setting, deletion, random cropping, shifting and re-quantization, respectively.

Next, we discuss the attack detection performance of this protocol. It is assumed that the illegal attacker Eve is unable to acquire any pre-shared initial keys $IK_{j}$. She can only obtain the bucket value sequences ${\left\{S_{B_{i}}\right\}}_{j}$ that are transmitted in the public channels. Therefore, exhaustive guesses about the pre-shared modulated patterns have to be made to acquire the correct CKs. The larger the signal dimension of $IK_{j}$, the lower the probability of deciphering. Although Eve cannot acquire any useful information from eavesdropped random bucket value sequences, she can still disrupt the CKD process by attacking these bucket value sequences. Without loss of generality, in the following experiment we used the bucket value sequence of User 4 as the target to attack and made comparisons between the results of the interactive superposed CKD protocol and our protocol. We tested a total of 10 types of common attacks: disordering, forging, sub-resampling, over-resampling, tampering, zero-setting, deletion, random cropping, shifting and re-quantization. All these attacks can be divided into two categories: global attacks (see Figure 6) and local attacks (see Figure 7). It should be noted that cutting off the transmission channels and hacking into computers are beyond the scope of consideration, because no protocols can withstand these attacks.

**(1) Global attacks: disordering, forging and resampling.** Assume that Eve can acquire the entire bucket value sequence of User 4. She can disrupt the original order of the entire sequence (disordering), completely fabricate a new sequence to replace the original one (forging) or resample the bucket value sequence by interpolation to obtain a new one. Here, for the resampling attack, we used bilinear interpolation to perform 95% sub-resampling and 105% over-resampling on the original bucket sequence. We knew all these attacks would cause serve damage to the recovered watermarks and make them unrecognizable in both the interactive superposed CKD protocol and our protocol. The recovered ghost images and their binarized results of the interactive superposed CKD protocol under global attacks are presented in Figure 6e1,e2,g1,g2,i1,i2,k1,k2, and the corresponding results of the proposed protocol under these attacks are given in Figure 6f1,f2,h1,h2,j1,j2,l1,l2. It can be seen that after binarization, the results of these two CKD protocols are completely disorganized. However, in our protocol, the legitimate user knows that a recognizable “watermark” label should be recovered in the watermark-embedding region under normal circumstances, according to which the attack self-detection can be carried out. While in the interactive superposed CKD protocol, users cannot perform self-detection of attacks.

**(2) Local attacks: tampering, zero-setting deletion, random cropping, shifting and re-quantization.** When Eve only acquires some part of the bucket value sequence, she can also perform local attacks to disrupt communication. Concretely, she can change partial bucket values to their adjacent values (tampering), replace them with zeros (zero-setting), simply erase them to make them disappear from the original sequence but without complement (deletion and random cropping), shift a portion of bucket values as a whole to other positions (shifting) or re-quantize the bucket values with a minimum unit (re-quantization). Here, the deletion attack will delete a continuous segment of the bucket value sequence, and the random cropping attack will randomly delete some bucket values. Both of them will cause a reduction in the total length of the bucket value sequence, but the legitimate user is not aware of it and will still use the modulated patterns in the original order for reconstruction. The re-quantization attack rounds the bucket values to the minimum unit, so the fluctuation trend of bucket values is roughly retained. The corresponding results of the interactive superposed CKD protocol and our protocol under these attacks (each with the same attack operations) can be found in Figure 7. It can be clearly seen that the numbers of wrong points in binarized images of the interactive superposed CKD protocol (see Figure 7a2,c2,e2,g2,i2,k2) are generally larger than those of our protocol (see Figure 7b2,d2,f2,h2,j2,l2), and the positions of wrong points in the former are randomly dispersed, while those in the latter are more concentrated in or around the watermark-embedding region. For the tampering attack, the wrong points started to appear in our protocol when we tampered with 20 bucket values. As for the zero-setting attack, only setting any bucket value to zero would cause wrong points to appear in the binarized image. Additionally, the number of wrong points will increase with the number of the bucket values being set to zero. For the deletion and random cropping attacks, deleting the values would directly destroy the one-to-one correspondence between the bucket values and modulated patterns after deletion of locations. The further the deletion positions are in the front of the bucket sequence, the greater the impact will be. Here, we kept the total numbers of deleted bucket values in both deletion and random cropping attacks the same: 10. As for the shifting attack, we shifted the 1000th to 1199th bucket values in the sequence back by 200 positions, which means 400 bucket values in total swapped their positions. For the re-quantization attack, since the magnitude of the recorded bucket values was in order of $10^{4}$, we set the minimum unit of quantization to 100. It is worth mentioning that for these local attack tests, we only present the results with minimal attacks that allowed the wrong points to start appearing, i.e., the maximum limits of these six types of local attacks that our protocol could withstand. When the aforementioned local attacks are more serious, the number of wrong points will be larger, and in our protocol these wrong points will always appear preferentially in the watermark-embedding region.

Thus, in our protocol, the local attacks can be easily detected by the legitimate users when they find the wrong points in their recovered binarized FPs. Once a user finds that there is one or more wrong point, all CKs distributed in this round of communication should be discarded immediately and a new round of CKD requires a reboot. In addition, since there is a one-to-one correspondence between “watermark” labels and users, which channel is under attack can be immediately determined according to the “watermark” label of the binarized image with wrong points. While in the interactive superposed CKD protocol, since each FP is purely random and does not have any watermark-embedding region, the user cannot determine whether there is an attack or locate the attacked channel according to the restored binarized image. Therefore, in addition to providing identity authentication, the watermarking technology in this protocol also adds an extra layer of protection and attack self-detection to the system.

**Figure 8:**
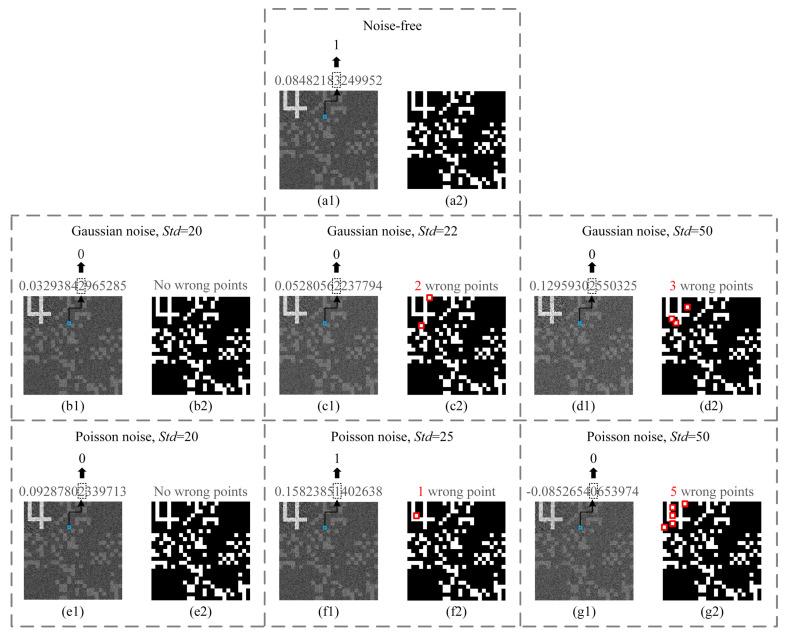
Performance comparisons between traditional CGI-based CKD protocol that utilizes the parity of decimals and our protocol, in the presence of additive white Gaussian noise and Poisson noise. (**a1,a2**) are the recovered ghost image with a blue pixel being marked over it (showing the cryptographic key extraction process in a traditional CGI-based CKD protocol while utilizing the parity of some digits after the decimal point of the gray value of the pixel at (60,90) of the ghost image) and the binarized result by using our protocol, under the noise-free condition. (**b1**,**b2**,**c1**,**c2**,**d1**,**d2**) and (**e1**,**e2**,**f1**,**f2**,**g1**,**g2**) are recovered ghost images and their binarized results by using the traditional CKD protocol and our protocol, under Gaussian noise with a standard deviation (Std) of 20, 22 or 50 and Poisson noise with a standard deviation of 20, 25 or 50, respectively. The red squares mark the wrong points.

Apart from the aforementioned attacks, the noise that may exist in the public channels will also have a certain impact on the transmitted bucket value sequence. Unlike the aforesaid deliberate attacks, the channel noise is generally independent and identically distributed. Here, we tested two types of common additive noise, i.e., white Gaussian noise and Poisson noise. We made some performance comparisons between traditional CGI-based CKD protocol [21,22] and our protocol. The former utilizes the parity of some particular digits after the decimal point of each gray value on the recovered ghost image to form a bit sequence as the distributed CK. In the following, we took the 8th decimal place of the grayscale value of the pixel at (60,90) of the ghost image for parity judgment, which can generate a bit, 0 or 1. The ghost image and binarized image under noise-free condition are given in Figure 8a1,a2 as a reference. Figure 8b1,b2,c1,c2,d1,d2 and 8e1,e2,f1,f2,g1,g2 present the results of the above two CKD protocols under Gaussian noise (with a standard deviation of 20, 22 and 50) and Poisson noise (with a standard deviation of 20, 25 and 50), respectively. It can be seen that the gray values are very sensitive to channel noise, which directly affects the values of the generated bits. In our protocol, when the standard deviation is less than 20, there are no wrong points in the binarized images (see Figure 8b2,e2) for both Gaussian and Poisson noise. When the standard deviation is greater than or equal to 22 under Gaussian noise, wrong points begin to appear first in the watermark-embedding region (see Figure 8c2,d2), and the number of wrong points increases with the standard deviation of noise. For Poisson noise, it is found that the wrong points begin to appear stably when the standard deviation is greater than or equal to 25 (see Figure 8f2,g2). This test proved that our protocol has a certain tolerance for noise existing in the public channels, whereas the traditional CKD protocol based on the parity of decimals does not.

## 5. Conclusions

In conclusion, here we proposed a CGI-based multi-party interactive CKD protocol over a public network, where a QR code image of Version 2 is used as the container of CKs and its functional region is treated as the watermark-embedding region. Since the QR code is used for CK extraction, the content of CKs will no longer be limited to binary streams, and can contain more information, such as video, pictures, hyperlinks and so on. In this protocol, the QR code image is split into multiple FPs, in each of which an independent “watermark” image label that corresponds to each user will be embedded in its functional region and then be treated as an original object image to be sampled. The modulated patterns are shared with the legitimate users in advance. The watermark-embedded FPs will be separately encrypted into random bucket value sequences via a CGI setup and be sent to users through public channels. This encryption process makes the “watermark” labels undetectable, and ensures the confidentiality and imperceptibility of the CKs. On the receiving end, each user can recover the ghost image and compute its binarized image. The content of the recovered “watermarks” will be used to perform users’ identity self-authentication. Thus, the watermarking technology used here helps to strengthen the protocol’s security. Furthermore, the spatial resolution of region patterns that locate in the watermark-embedding region of the FP can be different from that of non-watermark-embedding region, which can concentrate the wrong points more in or around the watermark-embedding region when an attack occurs. This makes the attack easier to detect. In addition, the use of cascade ablation in the intermediary realizes interactive joint authentication, adding second protection against illegal attacks. By scanning the returned cascade ablation result (recovered QR code), legitimate users can quickly acquire their distributed CKs. Both numerical simulations and optical experiments have demonstrated the feasibility of this protocol and its susceptibility to attacks. We have also performed some noise addition tests to show that this protocol has a certain tolerance for noise in the public channels. Therefore, this protocol may provide a new means of utilizing watermarking-based self-authentication and cascade-ablation-based interactive authentication in high-security CKD applications.

## Data Availability

Not applicable.
